# Impact of Pre-existing Type 2 Diabetes Mellitus and Cardiovascular Disease on Healthcare Resource Utilization and Costs in Patients With COVID-19

**DOI:** 10.36469/001c.92368

**Published:** 2024-04-19

**Authors:** Chi Nguyen, Christopher L. Crowe, Effie Kuti, Bonnie Donato, Rachel Djaraher, Leo Seman, Nancy Graeter, Thomas P. Power, Rinku Mehra, Vincent J. Willey

**Affiliations:** 1 Carelon Research, Wilmington, Delaware, USA; 2 Boehringer Ingelheim Pharmaceuticals, Inc., Ridgefield, Connecticut, USA; 3 Carelon Research, Wilmington Delaware, USA; 4 Carelon Medical Benefits Management, Chicago, Illinois, USA; 5 AmeriGroup, Virginia Beach, Virginia, USA

**Keywords:** COVID-19, cardiovascular diseases, healthcare costs, healthcare resource utilization, type 2 diabetes mellitus

## Abstract

**Background:** The economic burden associated with type 2 diabetes mellitus (T2DM) and concurrent cardiovascular disease (CVD) among patients with COVID-19 is unclear.

**Objective:** We compared healthcare resource utilization (HCRU) and costs in patients with COVID-19 and T2DM and CVD (T2DM + CVD), T2DM only, or neither T2DM nor CVD (T2DM/CVD).

**Methods:** A retrospective observational study in COVID-19 patients using data from the Healthcare Integrated Research Database (HIRD®) was conducted. Patients with COVID-19 were identified between March 1, 2020, and May 31, 2021, and followed from first diagnosis or positive lab test to the end of health plan enrollment, end of study period, or death. Patients were assigned one of 3 cohorts: pre-existing T2DM+CVD, T2DM only, or neither T2DM/CVD. Propensity score matching and multivariable analyses were performed to control for differences in baseline characteristics. Study outcomes included all-cause and COVID-19–related HCRU and costs.

**Results:** In all, 321 232 COVID-19 patients were identified (21 651 with T2DM + CVD, 28 184 with T2DM only, and 271 397 with neither T2DM/CVD). After matching, 6967 patients were in each group. Before matching, 46.0% of patients in the T2DM + CVD cohort were hospitalized for any cause, compared with 18.0% in the T2DM-only cohort and 6.3% in the neither T2DM/CVD cohort; the corresponding values after matching were 34.2%, 26.0%, and 21.2%. The proportion of patients with emergency department visits, telehealth visits, or use of skilled nursing facilities was higher in patients with COVID-19 and T2DM + CVD compared with the other cohorts. Average all-cause costs during follow-up were 12 324,7882, and $7277 per-patient-per-month after matching for patients with T2DM + CVD, T2DM-only, and neither T2DM/CVD, respectively. COVID-19–related costs contributed to 78%, 75%, and 64% of the overall costs, respectively. The multivariable model showed that per-patient-per-month all-cause costs for T2DM + CVD and T2DM-only were 54% and 21% higher, respectively, than those with neither T2DM/CVD after adjusting for residual confounding.

**Conclusion:** HCRU and costs in patients were incrementally higher with COVID-19 and pre-existing T2DM + CVD compared with those with T2DM-only and neither T2DM/CVD, even after accounting for baseline differences between groups, confirming that pre-existing T2DM + CVD is associated with increased HCRU and costs in COVID-19 patients, highlighting the importance of proactive management.

## BACKGROUND

The World Health Organization declared COVID-19 a global pandemic in March 2020. The disease has so far affected approximately 761 071 826 people and contributed to 6 879 677 deaths globally as of March 21, 2023.^1^ Diabetes is a common comorbidity, reported in approximately 5% to 36% of patients with COVID-19.^2–5^ The comorbidity burden and diabetes-related complications are expected to increase the risk of adverse outcomes and raise the healthcare resource utilization (HCRU) and costs among patients with COVID-19. Numerous studies have shown that patients with diabetes are at higher risk for developing severe COVID-19 and have a higher risk for increased mortality compared with those without diabetes.^6–9^ A study reported that the cost per hospital admission during the first wave of COVID-19 was remarkably higher for patients with type 2 diabetes mellitus (T2DM) compared with those without T2DM: EUR €25 018 for those with optimal glycemic levels, €46 130 for those with suboptimal glycemic levels compared with patients without diabetes (€16 993).^9^ In addition, a poor prognosis in patients with COVID-19 and diabetes is suggested to be linked to other comorbidities commonly present with diabetes mellitus, such as cardiovascular disease (CVD).^6^

Cardiovascular disease affects 32.2% of patients with T2DM globally and is a major cause of death and disability.^10^ Two separate meta-analyses (N = 65 484 and 4448 patients with COVID-19, respectively) have shown that pre-existing CVD was associated with a high risk of mortality and severe COVID-19.^11,12^ In a study in Bangladesh, the co-occurrence of CVD and diabetes in patients with COVID-19 was one of the main predictors of poor prognosis and mortality (odds ratio [OR]: 6.98; 95% CI: 4.21-7.34).^13^

The emerging evidence explains the adverse impact of both T2DM and CVD on COVID-19 outcomes.^6–8,11,12^ However, the impact of concurrent CVD+T2DM in patients with COVID-19 is evaluated in a limited number of studies^13^; in particular, the data regarding HCRU and costs are scarce. The current retrospective database study was performed to compare the economic burden, including all-cause and COVID-19–related HCRU and costs, in 3 COVID-19 patient cohorts—those with pre-existing T2DM + CVD, T2DM-only, and neither T2DM/CVD.

## METHODS

### Study Design and Settings

This was an observational cohort study of patients with COVID-19 using the data from the Healthcare Integrated Research Database (HIRD®), a proprietary insurance claims database. The HIRD is an administrative claims database of medical, pharmacy, and beneficiary eligibility and laboratory results data from commercial and Medicare Advantage health plans with members in all 50 US states. Mortality status was determined from a combination of the Social Security Death Index, claims-based inpatient discharge status, reasons for health plan disenrollment, and third-party obituary data. The HIRD is also linked to the 2017 American Community Survey for census block group-level socioeconomic status.

The data were used in full compliance with the relevant provisions of the Health Insurance Portability and Accountability Act. The study was conducted under the research provisions of Privacy Rule 45 CFR 164.514(e) and was exempt from Institutional Review Board review.

### Patient Identification

Patients with COVID-19, as defined by at least 1 COVID-19 medical claim (*International Classification of Diseases [ICD] Tenth Revision, Clinical Modification*: U0.71) in inpatient/emergency department (ED)/outpatient settings or at least 1 COVID-19 positive laboratory-confirmed test (either molecular or antigen test) between March 1, 2020, and May 31, 2021, were identified from the database (**Supplementary Figure S1**). The index date was defined as the earliest date of a medical claim with a COVID-19 diagnosis or a positive laboratory test (either molecular or antigen test) for COVID-19. Patients were at least 18 years old as of the index date and had continuous health plan enrollment for at least 12 months before the index date. They were excluded if they had type 1 diabetes mellitus (T1DM) before the index date. T1DM was defined as having at least 2 claims for T1DM on distinct dates and at least 1 claim for insulin or an insulin pump and no fill for non-insulin diabetes medications except metformin.

Patients were classified into 1 of 3 cohorts by the presence of T2DM and CVD before COVID-19 infection. The first cohort, defined as T2DM + CVD, consisted of patients with COVID-19 who had both T2DM and CVD before the index date. T2DM was defined as having at least 2 claims for T2DM on distinct dates or at least 1 claim for T2DM and at least 1 prescription fill for antidiabetic medications (including insulin). CVD was defined as having at least 1 claim in the inpatient/ED setting or at least 2 claims in the outpatient setting for any of the following cardiac diseases/events or vascular diseases: myocardial infarction, stroke, transient ischemic attack, unstable angina, other cerebrovascular diseases, peripheral artery disease, coronary revascularization procedure, other coronary heart diseases, and heart failure. The second cohort, defined as T2DM-only, consisted of patients with COVID-19 who had T2DM and no claims for CVD before the index date. The third cohort consisted of patients with COVID-19 with neither T2DM nor CVD, who had no claims with a diagnosis for T2DM or CVD before the index date. The ICD-10-CM codes are included in **Supplementary Table S1**. Patients were followed from the index date until health plan disenrollment, end of the study period, or death, whichever occurred first.

### Study Outcomes

The economic outcomes included all-cause and COVID-19–related HCRU and healthcare costs. The HCRU included inpatient hospitalization, ED visits, use of outpatient services, use of skilled nursing facilities, and frequency of pharmacy prescription fills. HCRU is presented as the number of patients with at least 1 encounter. The number of encounters and healthcare costs were reported as per patient per month (PPPM) to account for variable length of follow-up. In addition, average monthly healthcare costs of patients who were alive and not lost to follow-up were reported for up to 12 months following the index date after partitioning the follow-up duration into monthly intervals post-index. Total costs were defined as the sum of the medical and pharmacy costs for both the plan-paid (by all payers) and the patient-paid (sum of co-pay, coinsurance, and deductible) services. Healthcare medical encounters and costs were considered to be related to COVID-19 if the claims had a COVID-19 diagnosis code in the primary or secondary position. All costs were adjusted to 2020 US dollars based on the Consumer Price Index of the US Bureau of Labor Statistics.

### Statistical Analysis

Categorical variables were presented as frequency and percentage, while continuous variables were presented as mean ± SD and median (interquartile range [IQR]). A comparative analysis between the 3 cohorts was conducted using a one-way analysis of variance or Kruskal-Wallis test for continuous variables and χ^2^ tests or Fisher’s exact test for categorical variables, as applicable.

Propensity score matching was performed to balance the baseline characteristics between the 3 study cohorts. A propensity score for each patient was estimated by fitting a logistic regression model. The dependent variable was cohort status; the independent variables included patient age on the index date, sex, geographic region, Medicare Advantage enrollment, baseline Quan-Charlson Comorbidity Index, and socioeconomic status. Patients were matched on propensity score using a prespecified caliper of 0.05. They were also exactly matched on COVID-19 diagnosis month and year to account for the COVID-19 treatment guidelines and vaccination, which evolved during the pandemic. Two propensity-matched cohorts were identified: 1:1 propensity-matched cohorts of neither T2DM/CVD vs T2DM-only, and another 1:1 matched cohort of neither T2DM/CVD vs T2DM + CVD. The common patients of both cohorts were identified to form a final 1:1:1 matched cohort. The balance between the matched cohorts was assessed using standardized differences. A standardized difference of more than 0.2 would indicate the possibility of an imbalance between cohorts.^14^

After matching, generalized linear models using a log link function and γ distribution were used to examine the healthcare costs for the 3 study cohorts while adjusting for potentially remaining residual confounding.

## RESULTS

**Figure 1.**
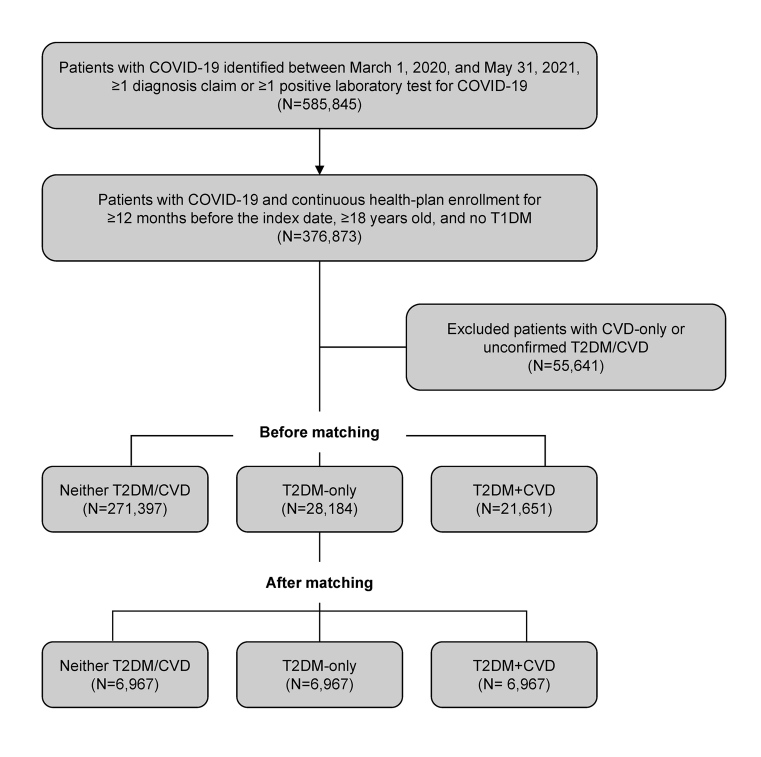
Flow Chart of the Study Population Abbreviations: CVD, cardiovascular disease; T1DM, type 1 diabetes mellitus; T2DM, type 2 diabetes mellitus.

From the database, initially, 585 845 individuals with a COVID-19 diagnosis claim or positive COVID-19 lab test were identified. Of these patients with COVID-19, 376 873 had continuous health plan enrollment for at least 12 months before the index date, were at least 18 years old, and had no T1DM (**Figure 1**). After excluding 55 641 patients (patients with CVD only, only 1 claim for T2DM without antidiabetics, or only 1 outpatient claim for CVD), there were 21 651 patients with pre-existing T2DM + CVD, 28 184 patients with T2DM-only, and 271 397 patients with neither T2DM/CVD. After propensity score matching, the final matched sample comprised 6967 patients in each of the 3 cohorts.

### Baseline Characteristics

The demographic and clinical characteristics are presented in **Table 1**. Before propensity score matching, patients with COVID-19 and pre-existing T2DM + CVD were significantly older on their index date than those with neither condition (69.9 ± 13.1 years, 54.5 ± 11.7 years, and 40.9 ± 14.5 years for patients with T2DM + CVD, T2DM-only, and neither T2DM/CVD, respectively; *P* < .001). Similarly, patients with COVID-19 and pre-existing T2DM + CVD had a greater comorbidity burden than those with neither condition. After propensity score matching, the patient demographics and comorbidity indexes of the 3 cohorts were well balanced. However, residual differences remained in several baseline clinical conditions, such as hypertension, obesity, and chronic kidney disease, which were more frequent in the T2DM + CVD cohort, whereas cancer was found more commonly in the T2DM-only cohort.

**Table 1.** Patient Demographics and Clinical Characteristics

**Variables**	**Before Matching**			**After Matching**			
	**Neither T2DM/⁠CVD** **(N = 271 397)**	**T2DM-⁠Only** **(N = 28 184)**	**T2DM + CVD** **(N = 21 651)**	**Neither T2DM/CVD (N = 6967)**	**T2DM-⁠Only (N = 6967)**	**T2DM+CVD (N = 6967)**	**Standardized Difference (T2DM-Only vs Neither, T2DM + CVD vs Neither)**
Follow-up duration (mo), mean (SD)	5.4 ± 3.0	5.5 ± 3.1	5.2 ± 3.4	5.4 ± 3.0	5.4 ± 3.0	5.3 ± 3.0	-0.01, -0.02
Demographics							
Age (y) on index date, mean (SD)	40.9 ± 14.5	54.5 ± 11.7	69.9 ± 13.1	63.6 ± 10.8	62.7 ± 10.2	62.7 ± 10.8	-0.08, -0.08
Male, n (%)	124 842 (46.0)	13 557 (48.1)	11 507 (53.2)	3833 (55.0)	774 (54.2)	3843 (55.2)	0.02, 0.00
Female, n (%)	146 555 (54)	14 627 (51.9)	10 144 (46.9)	3134 (45.0)	3193 (45.8)	3124 (44.8)	0.02, 0.00
Geographic region, n (%)							0.09, 0.15
West	47 941 (17.7)	4259 (15.1)	3105 (14.3)	789 (11.3)	914 (13.1)	769 (11.0)	
South	99 026 (36.5)	11 315 (40.2)	5898 (27.2)	2493 (35.8)	2679 (38.5)	2372 (34.0)	
Northeast	42 056 (15.5)	3833 (13.6)	4487 (20.7)	1144 (16.4)	1036 (14.9)	1251 (18.0)	
Midwest	81 865 (30.2)	8745 (31.0)	8154 (37.7)	2538 (36.4)	2331 (33.5)	2572 (36.9)	
Missing	509 (0.2)	32 (0.1)	≤10	≤10	≤10	≤10	
COVID-19 diagnosis date, n (%)							0.00, 0.00
March 2020–Oct. 2020	76 422 (28.2)	8013 (28.5)	6986 (32.3)	1787 (25.7)	1787 (25.7)	1787 (25.7)	
Nov. 2020–Feb. 2021	159 006 (58.6)	16 962 (60.2)	12 544 (57.9)	4524 (64.9)	4524 (64.9)	4524 (64.9)	
March 2021–May 2021	35 969 (13.3)	3209 (11.4)	2121 (9.8)	656 (9.5)	656 (9.5)	656 (9.5)	
Insurance type							-0.01, 0.07
Commercial health plan	262 347 (96.7)	24 042 (85.3)	8744 (40.4)	4763 (68.4)	4806 (69.0)	4545 (65.2)	
Medicare Advantage health plan	8062 (3.0)	3810 (13.5)	10 511 (48.6)	2204 (31.6)	2161 (31.0)	2422 (34.8)	
Medicare–other	988 (0.4)	332 (1.2)	2396 (11.1)	–	–	–	
Neighborhood-⁠level SES index score, mean (SD)	54.8 ± 6.1	53.0 ± 5.5	53.5 ± 5.9	53.1 ± 5.7	52.3 ± 5.4	53.1 ± 5.5	−0.14, 0.00
Baseline clinical characteristics							
Quan-Charlson comorbidity index, mean (SD)	0.2 ± 0.7	0.7 ± 1.2	2.7 ± 2.3	1.5 ± 1.7	1.5 ± 1.6	1.7 ± 2.0	0.01, 0.07
Clinical conditions, n (%)							
Hypertension	43 364 (16)	18 296 (64.9)	19 585 (90.5)	3306 (47.5)	5324 (76.4)	6034 (86.6)	0.62, 0.92
Obesity (BMI ≥30 kg/m^2^)	38 194 (14.1)	11 381 (40.4)	8442 (39.0)	1504 (21.6)	2718 (39.0)	3102 (44.5)	0.39, 0.50
Severe obesity (BMI ≥40 kg/m^2^)	12 959 (4.8)	5397 (19.2)	4175 (19.3)	438 (6.3)	154 (16.6)	1489 (21.4)	0.33, 0.45
Asthma	15 829 (5.8)	2511 (8.9)	2536 (11.7)	1123 (16.1)	865 (12.4)	781 (11.2)	−0.11, −0.14
Cancer	8138 (3.0)	1751 (6.2)	3302 (15.3)	1687 (24.2)	969 (13.9)	644 (9.2)	−0.26, −0.41
COPD	6123 (2.3)	1514 (5.4)	5389 (24.9)	1113 (16.0)	727 (10.4)	1182 (17.0)	−0.16, 0.03
Chronic kidney disease	2308 (0.9)	1682 (6.0)	6711 (31.0)	667 (9.6)	1019 (14.6)	1280 (18.4)	0.16, 0.26
Autoimmune diseases	5402 (2.0)	894 (3.2)	1245 (5.8)	461 (6.6)	311 (4.5)	330 (4.7)	−0.09, −0.08
End-stage renal disease	104 (0.0)	88 (0.3)	1012 (4.7)	14 (0.2)	40 (0.6)	209 (3.0)	0.06, 0.22
Organ transplant	389 (0.1)	127 (0.5)	343 (1.6)	49 (0.7)	55 (0.8)	104 (1.5)	0.01, 0.08
HIV/AIDS	580 (0.2)	99 (0.4)	69 (0.3)	135 (1.9)	60 (0.9)	20 (0.3)	-0.09, -0.16
Chronic liver disease	425 (0.2)	110 (0.4)	167 (0.8)	94 (1.3)	67 (1.0)	46 (0.7)	-0.04, -0.07

Before matching: all *P* values <.01; after matching: the standardized differences were reported for both T2DM vs neither and T2DM + CVD vs neither.

Abbreviations: BMI, body mass index; COPD, chronic obstructive pulmonary disease; CVD, cardiovascular disease; SES, socioeconomic status; T2DM, type 2 diabetes mellitus.

### Healthcare Resource Utilization Following COVID-19 Infection

Before propensity score matching, 46.8% of patients with COVID-19 and T2DM + CVD were hospitalized (all-cause), followed by 18.0% of those in the T2DM-only cohort, and 6.3% of those in the cohort of neither T2DM/CVD. COVID-19–related hospitalization followed a similar trend, and the proportions of patients hospitalized in the 3 cohorts were 38.4%, 15.2%, and 4.3%, respectively (**Supplementary Table S2**). After matching, 34.2%, 26.0%, and 21.2% of patients were hospitalized for any cause in the T2DM + CVD cohort, T2DM-only cohort, and in the cohort of neither T2DM/CVD, respectively (**Table 2**). COVID-19–related hospitalization followed a similar trend, and the highest percentage of hospitalized patients was seen in the T2DM + CVD cohort compared with patients from the other 2 cohorts after matching (**Table 2**).

**Table 2.** All-Cause and COVID-19–Related HCRU in Propensity-Matched Cohorts

	**All-Cause HCRU**				**COVID-19–Related HCRU**			
**Variables**	**Neither T2DM/⁠CVD (N = 6967)**	**T2DM-⁠Only (N = 6967)**	**T2DM+CVD (N = 6967)**	***P* Value**	**Neither T2DM/⁠CVD (N = 6967)**	**T2DM-⁠Only (N = 6967)**	**T2DM + CVD (N = 6967)**	***P* Value**
Duration of hospital stay, days	8.9 ± 10.5 6.0 (4.0-10.0)	9.9 ± 11.1 6.0 (4.0-11.0)	9.5 ± 10.5 6.0 (4.0-11.0)	<.001	9.9 ± 11.6 6.0 (4.0-11.0)	10.9 ± 11.9 7.0 (4.5-12.0)	10.9±12.4 7.0 (5.0-12.0)	.003
30-day readmissions	187 (12.7)	216 (11.9)	418 (17.5)	<.001	95 (7.9)	125 (8.0)	194 (10.1)	.042
ED visits	1420 (20.4)	1705 (24.5)	1991 (28.6)	<.001	905 (13.0)	1159 (16.6)	1153 (16.5)	<.001
Outpatient services	6749 (96.9)	6707 (96.3)	6685 (96.0)	.013	5166 (74.1)	5119 (73.5)	5022 (72.1)	.019
In-person physician-office visits	6088 (87.4)	6211 (89.1)	6151 (88.3)	.005	3187 (45.7)	3257 (46.7)	3003 (43.1)	<.001
No. of visits per month	0.9 ± 1.6	1.0 ± 1.6	1.1 ± 1.4	<.001	0.2 ± 1.1	0.2 ± 1.3	0.2±0.9	<.001
Telehealth visits	2802 (40.2)	3054 (43.8)	3366 (48.3)	<.001	2364 (33.9)	2637 (37.8)	2916 (41.9)	<.001
Durable medical equipment use	1106 (15.9)	1376 (19.8)	1830 (26.3)	<.001	226 (3.2)	347 (5.0)	304 (4.4)	<.001
Imaging	2443 (35.1)	2343 (33.6)	3119 (44.8)	<.001	117 (1.7)	132 (1.9)	172 (2.5)	.003
Medication and related services	2236 (32.1)	2358 (33.8)	2679 (38.5)	<.001	335 (4.8)	417 (6.0)	449 (6.4)	<.001
Physician–other services	2933 (42.1)	3232 (46.4)	3707 (53.2)	<.001	997 (14.3)	1063 (15.3)	1240 (17.8)	<.001
Procedures	2289 (32.9)	2276 (32.7)	2738 (39.3)	<.001	43 (0.6)	92 (1.3)	83 (1.2)	<.001
Physical therapy/⁠occupational therapy	744 (10.7)	703 (10.1)	995 (14.3)	<.001	67 (1.0)	101 (1.4)	164 (2.4)	<.001
Tests (lab)	4610 (66.2)	5233 (75.1)	5228 (75)	<.001	792 (11.4)	909 (13.0)	877 (12.6)	<.008
Tests (other)	1781 (25.6)	1911 (27.4)	2935 (42.1)	<.001	185 (2.7)	238 (3.4)	249 (3.6)	<.005
Outpatient–other	6022 (86.4)	5912 (84.9)	5893 (84.6)	.004	3547 (50.9)	3371 (48.4)	3192 (45.8)	<.001
Skilled nursing facility	189 (2.7)	192 (2.8)	360 (5.2)	<.001	125 (1.8)	107 (1.5)	226 (3.2)	<.001
Pharmacy prescription fills	6022 (86.4)	6350 (91.1)	6373 (91.5)	<.001	776 (11.1)	811 (11.6)	882 (12.7)	<.018
No. of fills per month	1.5 ± 1.7	2.2 ± 2.0	2.8 ± 2.3	<.001	0.0 ± 0.2	0.0 ± 0.3	0.1 ± 0.3	.026

Data are presented as mean ± SD or median (IQR) or n (%).

*P* value for comparison of 3 cohorts calculated using χ^2^ test or Fisher’s exact test for categorical variables and ANOVA or Kruskal-Wallis test for continuous variables.

Abbreviations: ANOVA, analysis of variance; CVD, cardiovascular disease; ED, emergency department; HCRU, healthcare resource utilization; IQR, interquartile range; T2DM, type 2 diabetes mellitus.

Before matching, 20.9% of patients with COVID-19 and T2DM + CVD were readmitted to the hospital (all-cause hospitalization) within 30 days of being discharged; a percentage that was considerably higher compared with 9.5% of patients with COVID-19 and T2DM-only, and 8.3% of patients with COVID-19 and neither T2DM/CVD. COVID-19–related 30-day readmission followed a similar trend, with the highest percentage of readmitted patients seen in the T2DM + CVD cohort compared with the other 2 cohorts (**Supplementary Table S2**). After matching, 17.5%, 11.9%, and 12.7% of patients were readmitted within 30 days (all-cause) in the T2DM + CVD, the T2DM-only, and the neither T2DM/CVD cohort, respectively. A similar pattern was seen for COVID-19–related 30-day readmission and the highest percentage of readmitted patients was observed in the T2DM + CVD cohort compared with the other 2 cohorts after matching (**Table 2**).

Before matching, the all-cause median duration of hospital stay was longer for patients with COVID-19 and pre-existing T2DM + CVD (7.0 days, IQR: 4.0-11.5 days), and for those with T2DM only (6.0 days, IQR: 4.0-10.0 days), compared with patients who had neither condition (5.0 days, IQR: 3.0-7.0 days). Similarly, the COVID-19–related median duration of hospital stay was longer among patients with COVID-19 and pre-existing T2DM + CVD (7.0 days, IQR: 5.0-13.0 days), and for those with T2DM-only (6.0 days, IQR: 4.0-11.0 days), compared with patients who had neither condition (6.0 days, IQR: 4.0-9.0 days) (**Supplementary Table S2**). After matching, the all-cause median duration of hospital stay was 6.0 days (IQR: 4.0-11.0 days) in patients with COVID-19 and T2DM + CVD, and in patients with COVID-19 and T2DM-only, and 6.0 days (IQR: 4.0-10.0 days) in patients with COVID-19 who had neither T2DM/CVD (**Table 2**). The COVID-19–related median duration of hospital stay in the 3 cohorts was 7.0 (IQR: 5.0-12.0 days), 7.0 (IQR: 4.5-12.0 days), and 6.0 (IQR: 4.0-11.0 days) days, respectively (**Table 2**).

The all-cause and COVID-19–related use of ED visits, telehealth visits, the number of in-person physician office visits PPPM, use of other physician services, use of skilled nursing facilities, and pharmacy prescription filling showed similar patterns, with the highest utilization observed in patients with COVID-19 and pre-existing T2DM + CVD compared with the other 2 cohorts before (**Supplementary Table S2**) and after (**Table 2**) propensity score matching analyses.

In summary, there was a trend for a higher all-cause and COVID-19–related utilization of healthcare resources among patients with COVID-19 and pre-existing T2DM + CVD, and in those with COVID-19 and T2DM-only, compared with patients with COVID-19 and neither T2DM/CVD, both before and after propensity score-matching analysis.

## Healthcare Costs Following COVID-19 Infection

**Figure 2.**
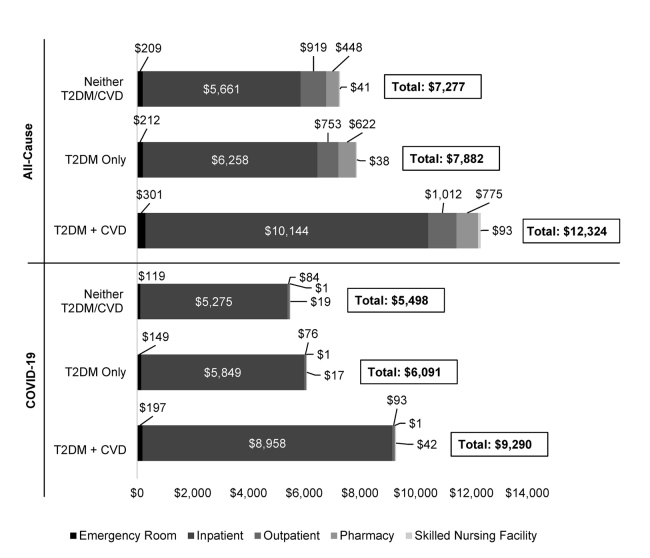
Mean Healthcare Costs Per-Patient-Per-Month After Propensity Score Matching All *P* values <.001 for comparison of the 3 cohorts. Individual costs may not sum to total due to rounding. Abbreviations: CVD, cardiovascular disease; T2DM, type 2 diabetes mellitus.

The average all-cause costs during follow-up, presented as PPPM, were US $14 790, $5717, and $1891 before matching (**Supplementary Figure S2**), and $12 324, $7882, and $7277 after matching (**Figure 2**), for patients with COVID-19 and pre-existing T2DM + CVD, COVID-19 and T2DM-only, and patients with COVID-19 and neither T2DM/CVD, respectively. The costs attributable to COVID-19 were 77.9%, 74.9%, and 63.7% of total all-cause costs before matching, and 75.4%, 77.3%, and 75.6% of total all-cause costs after matching for patients with pre-existing T2DM + CVD, T2DM-only, and patients with neither T2DM/CVD, respectively. Inpatient medical expenses accounted for most all-cause and COVID-19–related costs.

**Figure 3.**
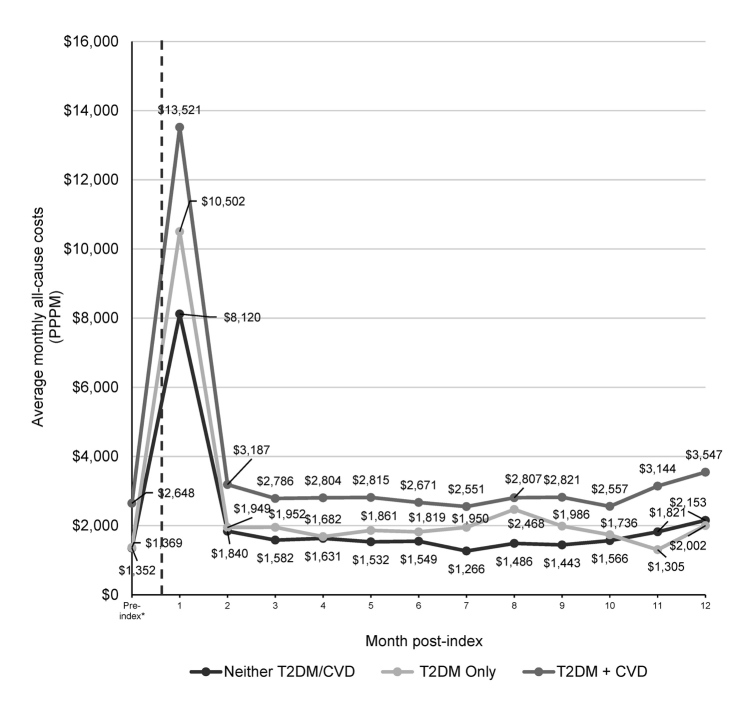
Mean Monthly All-Cause Costs After Propensity Score Matching ^a^Pre-index costs were calculated as the mean costs during the 12-month period before the index date; all *P* values <.001 for comparison of the 3 cohorts. *Vertical dotted line* represents the index date. Costs were adjusted to 2020 US dollars based on the Consumer Price Index. Abbreviations: CVD, cardiovascular disease; PPPM, per-patient-per-month; T2DM, type 2 diabetes mellitus.

After the multivariable adjustment, patients with COVID-19 and pre-existing T2DM + CVD had 54% higher PPPM costs compared with patients with COVID-19 who had neither T2DM/CVD (all-cause OR: 1.54; 95% CI: 1.44-1.63). Similarly, patients with COVID-19 and T2DM-only had 21% higher PPPM (all-cause OR: 1.21, 95% CI: 1.14-1.28; COVID-19–related OR: 1.28; 95% CI: 1.17-1.39) compared with those with COVID-19 and neither T2DM/CVD (**Supplementary Table S3**). Factors associated with increased PPPM cost included age at least 55 years, male sex, patients in the 3rd quartile of socioeconomic status, baseline Quan-Charlson Comorbidity Index at least 1, and comorbid chronic kidney disease or prior occurrence of acute respiratory distress syndrome/respiratory failure. Patients with COVID-19 in the south and midwest of the US had lower PPPM costs than those in the west of the country. Patients with COVID-19 who enrolled in a Medicare Advantage health plan had lower PPPM costs compared with those enrolled with a commercial health plan, most likely due to differences in contracted rates. Similarly, compared with patients with COVID-19 in the 1st quartile of socioeconomic status, patients in 2nd and 4th quartiles of socioeconomic status had lower PPPM costs. The average monthly healthcare costs increased during the first month after contracting COVID-19, then decreased but remained above pre-COVID-19 costs in before (*P* < .05) (**Supplementary Figure S3**) and after (*P* < .001) matching analyses (**Figure 3**). The average monthly healthcare costs were highest for patients with COVID-19 with pre-existing T2DM + CVD compared with those in the other 2 cohorts before (**Supplementary Figure S3**) and after (**Figure 3**) propensity score-matching analysis.

In summary, among the 3 cohorts of patients with COVID-19, those with pre-existing T2DM + CVD had higher all-cause and COVID-19–related healthcare costs compared with those with T2DM-only, and those with neither T2DM/CVD, before and after propensity score matching analysis.

## DISCUSSION

To our knowledge, this was the first study that evaluated HCRU and costs in patients with COVID-19 and pre-existing T2DM + CVD. Both before and after propensity score matching and multivariable adjustment, patients with COVID-19 and T2DM + CVD had higher HCRU and costs than patients with COVID-19 and neither T2DM/CVD.

In the present study, a higher percentage of patients were hospitalized or readmitted within 30 days of being discharged in the T2DM-only cohort compared with those in the cohort of neither T2DM/CVD. The healthcare costs were also higher for the patients in the T2DM-only cohort compared with those in the cohort of neither T2DM/CVD. These findings are in alignment with the adverse impact of diabetes on the HCRU and costs, which has been reported in the literature in patients with COVID-19.^9,15,16^ For example, Ko and colleagues estimated that individuals with diabetes were at greater than 3 times the risk for COVID-19–associated hospitalization compared to those without diabetes (adjusted risk ratio = 3.2; 95% CI: 2.5-4.1) across 12 states within the United States. These results were consistent with what Gregory et al observed in a large, single US healthcare system, with individuals with T2DM having greater than 3 times the odds of a COVID-19–associated hospitalization (adjusted odds ratio = 3.4; 95% CI: 2.5-4.6). However, more information was needed on the HCRU and cost burden associated with T2DM that is concurrently present with CVD in patients with COVID-19. The cost for disease management in patients with COVID-19 and T2DM was estimated by Bain and colleagues and shown to be higher in patients with pre-existing T2DM compared with those without T2DM.^9^ Our study showed an incremental increase in HCRU and costs in patients with COVID-19 and pre-existing T2DM + CVD compared with those with T2DM-only or neither T2DM/CVD.

The present study results confirm that pre-existing T2DM + CVD is associated with increased HCRU and costs in patients with COVID-19, highlighting the importance of proactive management of patients with COVID-19, which may include a comprehensive assessment of those with pre-existing T2DM + CVD presenting in the inpatient or outpatient setting, COVID-19 therapies suitable for this patient population, COVID-19 vaccination campaigns raising awareness for such patients, and optimal use of antidiabetic medications where appropriate. Glycemic control using sodium-glucose co-transporter-2 (SGLT-2) inhibitors and glucagon-like peptide-1 (GLP-1) receptor agonists are some of the recommended treatment approaches for patients with T2DM who have CVD comorbidity.^17^ A network meta-analysis showed that SGLT-2 inhibitors and GLP-1 receptor agonists effectively reduced the risk of cardiovascular and all-cause mortality in patients with T2DM + CVD.^18^ Such approaches could have a positive impact on cardiovascular outcomes in patients with COVID-19 who have pre-existing T2DM and concurrent CVD.^19^

### Limitations

This study has a few limitations that require consideration. There is a possibility of underestimation or overestimation of costs, depending on how they were being reported, due to selection bias, as patients who did not seek medical care (including those who were not tested or did not report COVID-19 infection, or those with asymptomatic infections) would not have available data and therefore would not have been included.

There were confounding factors that we could not control for, such as incomplete clinical information on vaccine administration, because such information was underreported in administrative claims data at the beginning of vaccination administration. However, the 3 cohorts were matched on their COVID-19 diagnosis timing to ensure that they were comparable in terms of their opportunity for exposure to vaccine and treatment protocols, which changed throughout the pandemic. Also, diabetes severity was not accounted for in the matching or multivariate models. In addition, the study estimated the direct costs only; it did not account for any additional indirect costs or investigate the costs of the long-term sequelae of COVID-19. Moreover, the documented hospital length of stay could be biased as patients who died were likely to have a shorter length of stay than those who survived.

Finally, this study was limited to patients with Medicare Advantage and commercial health insurance coverage, which may reduce the generalizability of these results to other populations, such as traditional Medicare and Medicaid patients and the uninsured patient population.

## CONCLUSIONS

The current study presents real-world data from a large commercial health insurance and Medicare Advantage population, estimating HCRU and costs for patients with COVID-19 and pre-existing T2DM + CVD. These patients have incrementally higher HCRU and costs compared with those with COVID-19 and T2DM-only, and those with COVID-19 and neither T2DM/CVD, even after accounting for baseline differences between groups. Our results highlight the need for healthcare systems and health insurance payers to adopt thorough assessment and management strategies to mitigate the economic burden associated with patients with COVID-19 who also have pre-existing T2DM + CVD.

## Supplementary Material

Online Supplementary Material

## References

[ref-292126] World Health Organization WHO coronavirus (COVID-19) dashboard.

[ref-292127] Abdelhafiz Ahmed H., Emmerton Demelza, Sinclair Alan J. (2021). Diabetes in COVID-19 pandemic-prevalence, patient characteristics and adverse outcomes. International Journal of Clinical Practice.

[ref-292128] Ugwueze Chidiebere V., Ezeokpo Basil Chukwuma, Nnolim Bede I., Agim Emmanuel A., Anikpo Nnamdi C., Onyekachi Kenechukwu E. (2020). COVID-19 and diabetes mellitus: the link and clinical implications. Dubai Diabetes and Endocrinology Journal.

[ref-292129] Li Gerui, Chen Ze, Lv Zhan, Li Hang, Chang Danqi, Lu Jinping (2021). Diabetes mellitus and COVID-19: associations and possible mechanisms. International Journal of Endocrinology.

[ref-292130] Corrao Salvatore, Pinelli Karen, Vacca Martina, Raspanti Massimo, Argano Christiano (2021). Type 2 diabetes mellitus and COVID-19: a narrative review. Frontiers in Endocrinology.

[ref-292131] Landstra Cyril P., de Koning Eelco J. P. (2021). COVID-19 and diabetes: understanding the interrelationship and risks for a severe course. Frontiers in Endocrinology.

[ref-292132] Hartmann-Boyce Jamie, Rees Karen, Perring James C., Kerneis Sven A., Morris Elizabeth M., Goyder Clare, Otunla Afolarin A., James Olivia E., Syam Nandana R., Seidu Samuel, Khunti Kamlesh (2021). Risks of and from SARS-CoV-2 infection and COVID-19 in people with diabetes: a systematic review of reviews. Diabetes Care.

[ref-292133] Gregg Edward W., Sophiea Marisa K., Weldegiorgis Misghina (2021). Diabetes and COVID-19: population impact 18 months into the pandemic. Diabetes Care.

[ref-292134] Bain Stephen C., Czernichow Sebastien, Bøgelund Mette, Madsen Maria Elmegaard, Yssing Cecilie, McMillan Annabell Cajus, Hvid Christian, Hettiarachchige Nadilka, Panton Ulrik Haagen (2021). Costs of COVID-19 pandemic associated with diabetes in Europe: a health care cost model. Current Medical Research and Opinion.

[ref-292135] Einarson Thomas R., Acs Annabel, Ludwig Craig, Panton Ulrik H. (2018). Prevalence of cardiovascular disease in type 2 diabetes: a systematic literature review of scientific evidence from across the world in 2007–2017. Cardiovascular Diabetology.

[ref-292136] Pranata Raymond, Huang Ian, Lim Michael Anthonius, Wahjoepramono Eka Julianta, July Julius (2020). Impact of cerebrovascular and cardiovascular diseases on mortality and severity of COVID-19–systematic review, meta-analysis, and meta-regression. Journal of Stroke and Cerebrovascular Diseases.

[ref-292137] Ssentongo P., Ssentongo A.E., Heilbrunn E.S., Ba D.M., Chinchilli V.M. (2020). Association of cardiovascular disease and 10 other pre-existing comorbidities with COVID-19 mortality: a systematic review and meta-analysis. PLoS One.

[ref-292138] Sharif Nadim, Ahmed Shamsun Nahar, Opu Rubayet Rayhan, Tani Mahmuda Rahman, Dewan Dolly, Daullah Muktasid Ud, Shanto Rakibul Islam, Parvez Anowar Khasru, Talukder Ali Azam, Dey Shuvra Kanti (2021). Prevalence and impact of diabetes and cardiovascular disease on clinical outcome among patients with COVID-19 in Bangladesh. Diabetes & Metabolic Syndrome: Clinical Research & Reviews.

[ref-292139] Faraone S.V. (2008). Interpreting estimates of treatment effects: implications for managed care. P T.

[ref-292140] Ko J.Y., Danielson M.L., Town M.. (2021). Risk factors for coronavirus disease 2019 (COVID-19)-associated hospitalization: COVID-19-associated hospitalization surveillance network and behavioral risk factor surveillance system. Clin Infect Dis.

[ref-292141] Gregory Justin M., Slaughter James C., Duffus Sara H., Smith T. Jordan, LeStourgeon Lauren M., Jaser Sarah S., McCoy Allison B., Luther James M., Giovannetti Erin R., Boeder Schafer, Pettus Jeremy H., Moore Daniel J. (2021). COVID-19 severity is tripled in the diabetes community: a prospective analysis of the pandemic’s impact in type 1 and type 2 diabetes. Diabetes Care.

[ref-292142] American Diabetes Association (2021). 9. Pharmacologic approaches to glycemic treatment: Standards of Medical Care in Diabetes-2021. Diabetes Care.

[ref-292143] Kanie T., Mizuno A., Takaoka Y.. (2021). Dipeptidyl peptidase-4 inhibitors, glucagon-like peptide 1 receptor agonists and sodium-glucose co-transporter-2 inhibitors for people with cardiovascular disease: a network meta-analysis. Cochrane Database Syst Rev.

[ref-292144] Kahkoska Anna R., Abrahamsen Trine Julie, Alexander G. Caleb, Bennett Tellen D., Chute Christopher G., Haendel Melissa A., Klein Klara R., Mehta Hemalkumar, Miller Joshua D., Moffitt Richard A., Stürmer Til, Kvist Kajsa, Buse John B., Duong Tim Q., N3C Consortium (2021). Association between glucagon-like peptide 1 receptor agonist and sodium-glucose cotransporter 2 inhibitor use and COVID-19 outcomes. Diabetes Care.

