# Supplementary material for: Impact of Pre-existing Type 2 Diabetes Mellitus and Cardiovascular Disease on Healthcare Resource Utilization and Costs in Patients With COVID-19
Source: J Health Econ Outcomes Res. 2024 Apr 19;11(1):112–21. doi: 10.36469/001c.92368 (PMC11110887; doi:10.36469/001c.92368)
Supplement: Online Supplementary Material [file jheor_2024_11_1_92368_224680.pdf]

Journal of Health Economics and Outcomes Research

Online Supplementary Material

Impact of Pre-existing Type 2 Diabetes Mellitus and Cardiovascular Disease on Healthcare Resource Utilization and Costs in Patients With COVID-19. *JHEOR*. 2024;11(1):112-121. doi:10.36469/jheor.2024.92368

Table S1: ICD Codes Used to Identify Diabetes, Cardiovascular Diseases, Quan–Charlson Comorbidity Index

Table S2. All-Cause and COVID-19–Related HCRU Before Propensity Score Matching

Table S3. All-Cause and COVID-19–Related Total Per-Patient-Per-Month Costs in Propensity Score–Matched Cohorts After Multivariable Adjustment

Figure S1. Study Design Diagram

Figure S2. Mean Healthcare Costs Per Patient Per Month Before Propensity Score Matching

Figure S3. Average Monthly All-Cause Costs After COVID-19 Infection Before Propensity Score Matching

This supplementary material has been provided by the authors to give readers additional information about their work.

This is an open-access article distributed under the terms of the Creative Commons Attribution 4.0 International License (CCBY-4.0). View this license's legal deed at http://creativecommons.org/licenses/by/4.0 and legal code at http://creativecommons.org/licenses/by/4.0/legalcode for more information.

Table S1. ICD Codes Used to Identify Diabetes, Cardiovascular Diseases, and Quan-Charlson Comorbidity Outcomes

**Codes to Identify Diabetes and CVD**			
	**ICD-9-CM**	**ICD-10-CM**	**Other Codes**
Diabetes			
Type 1 diabetes	250.x1, 250.x3	E10.%	
Type 2 diabetes	250.x0, 250.x2	E11.%	
CVD			
Myocardial infarction (new and old)	410.xx, 412.xx	I21.% , I22.0, I22.1, I22.2, I22.8, I22.9, I23.%, I25.2	
Unstable angina	411.1x	I20.0, I25.110, I25.700, I25.710, I25.720, I25.730, I25.750, I25.760, I25.790	
Stroke	430.xx -431.xx, 433.x1, 434. x1, 436.xx	I60%, I62%, I63%	
Transient ischemic attack	435.0, 435.1, 435.2, 435.3, 435.8, 435.9,<br>433.x0, V12.54	G45%, Z86.73	
Other cerebrovascular disease	437.xx, 438.xx, 432.xx	I65%, I67%, I66%, G46%	
Peripheral vascular/artery disease	440.xx, 443.9x	I70%, I73.9%	
Coronary revascularization procedure			ICD-9-CM procedure codes: 00.66, 36.09, 36.07, 36.06, 36.1x36.2x<br><br>ICD-10-CM procedure codes: 027%, 02C%, 021%<br><br>CPT codes: 92980, 92982, 92984, 92995, 92996, 92920, 92924, 92980, 92981, 92928, 92933, 92937, 92941, 92943, 33510-33516, 33517-33523, 33530, 33533-33536, 33572, 92921, 92925, 92929, 92934, 92938, 92944, 92973<br><br>HCPCS codes: C1874, C1875, C1876, C1877, G0290, G0291, S2205-S2209
Other coronary heart disease	413.xx-414.xx, 411.xx, 429.2x	I20.1, I20.8, I20.9, I24.0, I24.1, I24.8, I25.10, 125.111, I25.118, I25.119, I25.3, I25.41, I25.42, I25.7% (except codes with "0" in 6th position), I25.810, I25.811, I25.812, I25.82, I25.83, I25.84, I25.89, I25.9	
Heart failure	398.91, 402.01, 402.11, 402.91, 404.01, 404.03, 404.11, 404.13, 404.91, 404.93, 414.8, 425.11, 425.18, 425.4, 425.5, 425.9, 428.xx	I09.81, I11.0, I13.0, I13.2, I25.5, I42.0, I42.1, I42.2, I42.5, I42.6, I42.7, I42.8, I42.9, I50.%	
**Codes to identify clinical outcomes**			
	**ICD-10-CM Code**	**Revenue Code**	**CPT Code**
Intensive care unit		Any hospital admission with one or more of the following revenue codes: 200, 201, 202, 203, 204, 206, 207, 208, 209, 210, 211, 212, 213, 214 and 219	

Table S1. ICD Codes Used to Identify Diabetes, Cardiovascular Diseases, and Quan-Charlson Comorbidity Outcomes

Intubation and mechanical ventilation	ICD-10-CM procedure codes: 5A1935Z, 5A1945Z, 5A1955Z, 5A09357, 5A09358, 5A09359, 5A0935B, 5A0935Z, 5A09457, 5A09458, 5A09459, 5A0945B, 5A0945Z, 5A09557, 5A09558, 5A09559, 5A0955B, 5A0955Z		94002-94003
Extracorporeal membrane oxygenation	ICD-10-CM procedure codes: 5A1522F, 5A1522G, 5A1522H, 5A15223		33946-33949, 33951-33956, 33957, 33958, 33959, 33962, 33963, 33964, 33965, 33966, 33969, 33984, 33985, 33986, 33987, 33988, 33989
Acute respiratory distress syndrome/respiratory failure	ICD-10-CM diagnosis code: J80%, J96.%, R09.2		
Pulmonary embolism	ICD-10-CM diagnosis code: I26%, z86.711		
Deep vein thrombosis	ICD-10-CM diagnosis code: I80.%, I81.%, I82.%		
Ischemic stroke	ICD-10-CM diangosis code: I63.%		
New myocardial infarction	ICD-10-CM diagnosis code: I21.%, I22.0, I22.1, I22.2, I22.8, I22.9, I23.%		
Myocarditis	ICID-10-CM diagnosis code: I40.9, I41.%, I40.8, I40.1, I51.4		
Acute kidney failure	ICD-10-CM diagnosis code: N17%		
Use of home oxygen			HCPCS codes: A4606, A4615, A4616, A4617, A4619, A4620 E0424, E0425, E0430, E0431, E0433, E0434, E0435, E0439, E0440, E0441, E0442, E0443, E0444, E0445, E0447, E0455, E0555, E0580, E1352, E1353, E1354, E1355, E1356, E1357, E1358, E1390, E1391, E1392, K0738, K0740, S8120, S8121
**Quan-Charlson Comorbidity Index**			
**Comorbidities**	**ICD-9-CM**	**ICD-10-CM**	
Myocardial infarction	410.x, 412.x	I21.x, I22.x, I25.2	
Congestive heart failure	398.91, 402.01, 402.11, 402.91, 404.01, 404.03, 404.11, 404.13, 404.91, 404.93, 425.4-425.9, 428.x	I09.9, I11.0, I13.0, I13.2, I25.5, I42.0, I42.5-I42.9, I43.x, I50.x, P29.0	
Peripheral vascular disease	093.0, 437.3, 440.x, 441.x, 443.1-443.9, 447.1, 557.1, 557.9, V43.4	I70.x, I71.x, I73.1, I73.8, I73.9, I77.1, I79.0, I79.2, K55.1, K55.8, K55.9, Z95.8, Z95.9	
Cerebrovascular disease	362.34, 430.x-438.x	G45.x, G46.x, H34.0, I60.x-I69.x	
Dementia	290.x, 294.1, 331.2	F00.x-F03.x, F05.1, G30.x, G31.1	
Chronic pulmonary disease	416.8, 416.9, 490.x-505.x, 506.4, 508.1, 508.8	I27.8, I27.9, J40.%-J47.%, J60.%-J67.%, J68.4, J70.1, J70.3	

Table S1. ICD Codes Used to Identify Diabetes, Cardiovascular Diseases, and Quan-Charlson Comorbidity Outcomes

Rheumatic disease	446.5, 710.0-710.4, 714.0-714.2, 714.8, 725.x	M05.%, M06.%, M31.5, M32.%-M34.%, M35.1, M35.3, M36.0
Peptic ulcer disease	531.x-534.x	K25.%-K28.%
Mild liver disease	070.22, 070.23, 070.32, 070.33, 070.44, 070.54, 070.6, 070.9, 570.x, 571.x, 73.3, 573.4, 573.8, 573.9, V42.7	B18.%, K70.0-K70.3, K70.9, K71.3-K71.5, K71.7, K73.%, K74.%, K76.0, K76.2-K76.4, K76.8, K76.9, Z94.4
Diabetes without chronic complication	250.0-250.3, 250.8, 250.9	E10.0, E10.1, E10.6, E10.8, E10.9, E11.0, E11.1, E11.6, E11.8, E11.9, E12.0, E12.1, E12.6, E12.8, E12.9, E13.0, E13.1, E13.6, E13.8, E13.9, E14.0, E14.1, E14.6, E14.8, E14.9
Diabetes with chronic complication	250.4-250.7	E10.2-E10.5, E10.7, E11.2-E11.5, E11.7, E12.2-E12.5, E12.7, E13.2-E13.5, E13.7, E14.2-E14.5, E14.7
Hemiplegia or paraplegia	334.1, 342.x, 343.x, 344.0-344.6, 344.9	G04.1, G11.4, G80.1, G80.2, G81.%, G82.%, G83.0-G83.4, G83.9
Renal disease	403.01, 403.11, 403.91, 404.02, 404.03, 404.12, 404.13, 404.92, 404.93, 582.x, 583.0-583.7, 585.x, 586.x, 588.0, V42.0, V45.1, V56.x	I12.0, I13.1, N03.2-N03.7, N05.2-N05.7, N18.%, N19.%, N25.0, Z49.0-Z49.2, Z94.0, Z99.2
Any malignancy, including leukemia and lymphoma	140.x-172.x, 174.x-195.8, 200.x-208.x, 238.6	C00.%-C26.%, C30.%-C34.%, C37.%-C41.%, C43.%, C45.%-C58.%, C60.%-C76.%, C81.%-C85.%, C88.%, C90.%-C97.%
Moderate or severe liver disease	456.0-456.2, 572.2-572.8	I85.0, I85.9, I86.4, I98.2, K70.4, K71.1, K72.1, K72.9, K76.5, K76.6, K76.7
Metastatic solid tumor	196.x-199.x	C77.%-C80.%
HIV/AIDS	042.x-044.x	B20.%-B22.%, B24.%
**Codes to identify other baseline comorbid conditions**		
	**ICD-10-CM diagnosis code**	
Asthma	J45.%	
Autoimmune diseases	Rheumatoid arthritis: M05.%, M06.%<br>Psoriatic arthritis: L40.5%<br>Lupus: M32.%<br>Ulcerative colitis: K51.%<br>Crohn's disease: K50.%	
Cancer	C00.0-C96.Z	
Chronic kidney disease	N18.%	
Chronic liver disease	B18.%, K72.1%, K73.0	
Chronic obstructive pulmonary disease	J40.%, J41.%, J42.%, J43.%, J44.%	
End-stage renal disease	N18.6	
Hypertension	I10 (uncomplicated); I11.%, I12.%, I13.%, I15.% (complicated)	
Obesity	E66.%, Z68.%	
Severe obesity (BMI ≥40)	E66.01, E66.2, Z68.41,,Z68.42, Z68.43, Z68.44, Z68.45	
Organ transplant	Z94.%	
Pregnancy	O00.%-O9A.%	
Tobacco use	F17.2%	

Table S1. ICD Codes Used to Identify Diabetes, Cardiovascular Diseases, and Quan-Charlson Comorbidity Outcomes

**Codes to identify other medications and COVID-19 treatment/vaccine**
Medications will be queried from the claims database using proprietary Generic Product Identifier codes, HCPCS codes, and ICD-10-CM procedure codes.<br>COVID-19 vaccine: CPT codes: 0001A, 0002A, 0011A, 0012A, 0021A, 0022A, 0031A, 91300, 91301, 91302, 91303;<br>CVX codes: 207, 208, 210, 212; NDC codes: 00310122210, 0310122210, 59267100001, 59267100002, 59267100003, 5926710001, 59676058005, 59676058015, 5967658005, 80777027310, 80777027399, 8077727310

Abbreviations: BMI, body mass index; CPT, Current Procedural Terminology; CVX, vaccine-administered code set; HCPCS, Healthcare Common Procedure Coding System; ICD-9/10-CM, *International Classification of Diseases, Ninth/Tenth Revision, Clinical Modification;* NDC, National Drug Code; QCI, Quan-Charlson Comorbidity Index.

Table S2. All-Cause and COVID-19–Related HCRU Before Propensity Score Matching

	All-cause HCRU				COVID-19–Related HCRU			
**Variables**	**Neither T2DM/ CVD (N = 271 397)**	**T2DM-Only (N = 28 184)**	**T2DM+CVD (N = 21 651)**	***P* Value**	**Neither T2DM/ CVD (N = 271 397)**	**T2DM-Only (N = 28 184)**	**T2DM+CVD (N = 21 651)**	***P* Value**
Inpatient admissions	17 105 (6.3)	5085 (18.0)	10 140 (46.8)	<.001	11 582 (4.3)	4284 (15.2)	8 321 (38.4)	<.001
LOS, mean (SD), median	7.3 ± 10.1<br>5.0 (3.0-7.0)	9.4 ± 11.8<br>6.0 (4.010.0)	9.9 ± 10.8<br>7.0 (4.0-11.5)	<.001	8.8 ± 11.9<br>6.0 (4.0-9.0)	10.3 ± 12.9<br>6.0 (4.0-11.0)	11.2 ± 12.6<br>7.0 (5.0-13.0)	<.001
30-day readmissions	1417 (8.3)	484 (9.5)	2119 (20.9)	<.001	628 (5.4)	255 (6.0)	1044 (12.6)	<.001
ED visits	38 176 (14.1)	6327 (22.5)	6230 (28.8)	<.001	22 870 (8.4)	4213 (15)	3274 (15.1)	<.001
Outpatient services	265 067 (97.7)	27 459 (97.4)	20 022 (92.5)	<.001	200 656 (73.9)	20 897 (74.1)	14 525 (67.1)	<.001
Physician in-person office visits	237 478 (87.5)	25 642 (91.0)	16 906 (78.1)	<.001	140 784 (51.9)	14 278 (50.7)	7063 (32.6)	<.001
No. of visits per month	0.7 ± 1.4	0.9 ± 1.5	1.0 ± 1.4	<.001	0.3 ± 1.1	0.3 ± 1.1	0.2 ± 0.8	<.001
Telehealth visits	92 206 (34.0)	12 294 (43.6)	10 170 (47.0)	<.001	73 407 (27.1)	10 342 (36.7)	8947 (41.3)	<.001
Durable medical equipment use	18 739 (6.9)	4498 (16.0)	6487 (30.0)	<.001	2279 (0.8)	932 (3.3)	1080 (5.0)	<.001
Imaging	58 125 (21.4)	8375 (29.7)	9874 (45.6)	<.001	2883 (1.1)	398 (1.4)	577 (2.7)	<.001
Medication and related services	52 733 (19.4)	8470 (30.1)	7898 (36.5)	<.001	6207 (2.3)	1513 (5.4)	1153 (5.3)	<.001
Physician–other services	68 584 (25.3)	11 021 (39.1)	13 283 (61.4)	<.001	19 807 (7.3)	3493 (12.4)	4940 (22.8)	<.001
Procedures	49 967 (18.4)	7485 (26.6)	9281 (42.9)	<.001	1239 (0.5)	245 (0.9)	261 (1.2)	<.001
Physical therapy/occupational therapy	21 314 (7.9)	2656 (9.4)	3788 (17.5)	<.001	494 (0.2)	231 (0.8)	701 (3.2)	<.001
Tests (lab)	140 136 (51.6)	20 187 (71.6)	14 795 (68.3)	<.001	28 552 (10.5)	3433 (12.2)	2411 (11.1)	<.001
Tests (other)	37 209 (13.7)	6642 (23.6)	9176 (42.4)	<.001	4965 (1.8)	783 (2.8)	819 (3.8)	<.001
Outpatient–others	241 554 (89.0)	24 253 (86.1)	17 132 (79.1)	<.001	147 053 (54.2)	14 057 (49.9)	8435 (39.0)	<.001
Skilled nursing facility	530 (0.2)	318 (1.1)	2704 (12.5)	<.001	332 (0.1)	187 (0.7)	1707 (7.9)	<.001
Pharmacy prescription fills	194 218 (71.6)	25 422 (90.2)	19 001 (87.8)	<.001	12 103 (4.5)	2719 (9.7)	2704 (12.5)	<.001
No. of fills per month	0.8 ± 1.3	2.0 ± 1.9	2.9 ± 2.7	<.001	0.0 ± 0.2	0.0 ± 0.4	0.1 ± 0.4	<.001

Data are presented as mean ± SD or median (IQR) or n (%). *P* value for comparison of 3 cohorts calculated using χ^2 test or Fisher's exact test for categorical variables and ANOVA or Kruskal-Wallis test for continuous variables.
Abbreviations: CVD, cardiovascular disease; ED, emergency department; HCRU, healthcare resource utilization; IQR, interquartile range; LOS, length of stay; T2DM, type 2 diabetes mellitus.

Table S3. All-Cause and COVID-19–Related Total Per-Patient-Per-Month Costs in Propensity Score–Matched Cohorts After Multivariable Adjustment

	All-Cause Total PPPM Costs				COVID-19–Related Total PPPM Costs			
	Exponent of Estimate	95% CI		*P* Value[a]	Exponent of Estimate	95% CI		*P* Value[a]
		Lower	Upper			Lower	Upper	
Study cohort								
Neither T2DM/CVD (reference)	–	–	–	–	–	–	–	–
T2DM-only	1.21	1.14	1.28	<.001	1.28	1.17	1.39	<.001
T2DM + CVD	1.54	1.44	1.63	<.001	1.65	1.51	1.80	<.001
Covariates								
Baseline conditions (yes/no)								
Hypertension	0.99	0.94	1.05	.850	0.99	0.92	1.08	.859
Obesity (BMI ≥30 kg/m2)	1.02	0.97	1.08	.400	1.00	0.93	1.08	.975
Asthma	1.01	0.94	1.09	.706	1.04	0.94	1.15	.481
Cancer	0.98	0.91	1.06	.616	0.91	0.81	1.01	.076
COPD	1.08	1.01	1.16	.027	1.09	0.99	1.21	.082
Chronic kidney disease	1.52	1.41	1.63	<.001	1.78	1.60	1.98	<.001
Autoimmune disease	1.11	1.00	1.23	.049	0.99	0.86	1.16	.936
Other baseline all-cause clinical conditions (yes/no)								
ARDS/respiratory failure	1.59	1.39	1.82	<.001	2.02	1.67	2.45	<.001
Venous thromboembolism	0.85	0.75	0.97	.012	0.80	0.67	0.96	.015
Acute kidney failure	0.95	0.84	1.06	.354	0.90	0.76	1.07	.232
Baseline HCRU								
All-cause inpatient admission (yes/no)	0.93	0.86	1.01	.081	0.96	0.85	1.07	.465
All-cause total PPPM costs/100	1.01	1.01	1.01	<.001	1.00	1.00	1.01	<.001
Demographics								
Age on index date (years)								
18-54 (reference)	–	–	–	–	–	–	–	–
55-64	1.63	1.53	1.74	<.001	2.05	1.87	2.24	<.001
65-75	2.82	2.61	3.05	<.001	4.05	3.62	4.52	<.001
≥75	4.24	3.86	4.65	<.001	6.49	5.68	7.42	<.001
Sex								
Female (reference)	–	–	–	–	–	–	–	–
Male	1.56	1.49	1.63	<.001	1.85	1.73	1.99	<.001
Region on index date								
West (reference)	–	–	–	–	–	–	–	–
South	0.82	0.75	0.88	<.001	0.72	0.64	0.81	<.001
Northeast	0.89	0.81	0.97	.008	0.75	0.66	0.85	<.001
Midwest	0.75	0.69	0.81	<.001	0.65	0.58	0.74	<.001
Missing	0.78	0.32	1.92	.596	0.33	0.09	1.18	.089
Health plan type								
Health maintenance organization (reference)	–	–	–	–	–	–	–	–
Preferred provider organization	0.84	0.80	0.89	<.001	0.80	0.73	0.87	<.001

Consumer-directed health plan	0.84	0.78	0.92	<.001	0.85	0.75	0.96	.007
Other	0.03	0.02	0.05	<.001	0.00	0.00	0.01	<.001
Insurance type								
Commercial health plan (reference)	–	–	–	–	–	–	–	–
Medicare Advantage	0.92	0.85	0.99	.027	0.97	0.87	1.08	.600
Quartile distribution of SES								
1st quartile (lowest)(reference)	–	–	–	–	–	–	–	–
2nd quartile	0.93	0.87	0.99	.018	0.92	0.84	1.00	.052
3rd quartile	1.17	1.10	1.25	<.001	1.23	1.12	1.35	<.001
4th quartile (highest)	0.80	0.74	0.85	<.001	0.72	0.65	0.80	<.001
Missing	1.05	0.85	1.30	.641	1.04	0.77	1.40	.806
Baseline QCI								
0 (reference)	–	–	–	–	–	–	–	–
1	1.34	1.26	1.43	<.001	1.35	1.23	1.48	<.001
2	1.27	1.18	1.36	<.001	1.23	1.11	1.37	<.001
3+	1.88	1.72	2.05	<.001	1.88	1.66	2.13	<.001

Data are presented as mean ± SD or n (%).
[a]*P* values obtained from a generalized linear model using a log-link function and gamma distribution. Model sample size: 20,901
Abbreviations: ARDS, acute respiratory distress syndrome; BMI, body mass index; CI, confidence interval; COPD, chronic obstructive pulmonary disease; CVD, cardiovascular disease; HCRU, health care-resource utilization; PPPM, per patient per month; QCI, Quan-Charlson comorbidity index; SES, socioeconomic status; T2DM, type 2 diabetes mellitus.

Figure S1. Study Design Diagram

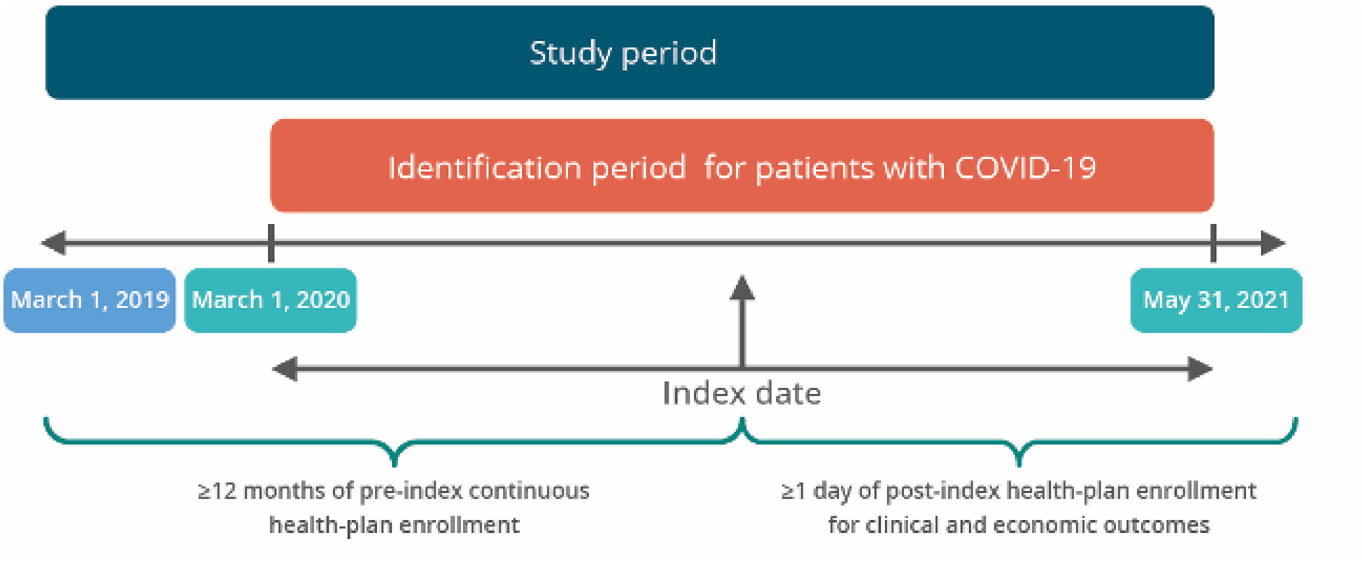

Figure S2. Mean Health Care Costs Per Patient Per Month Before Propensity Score Matching

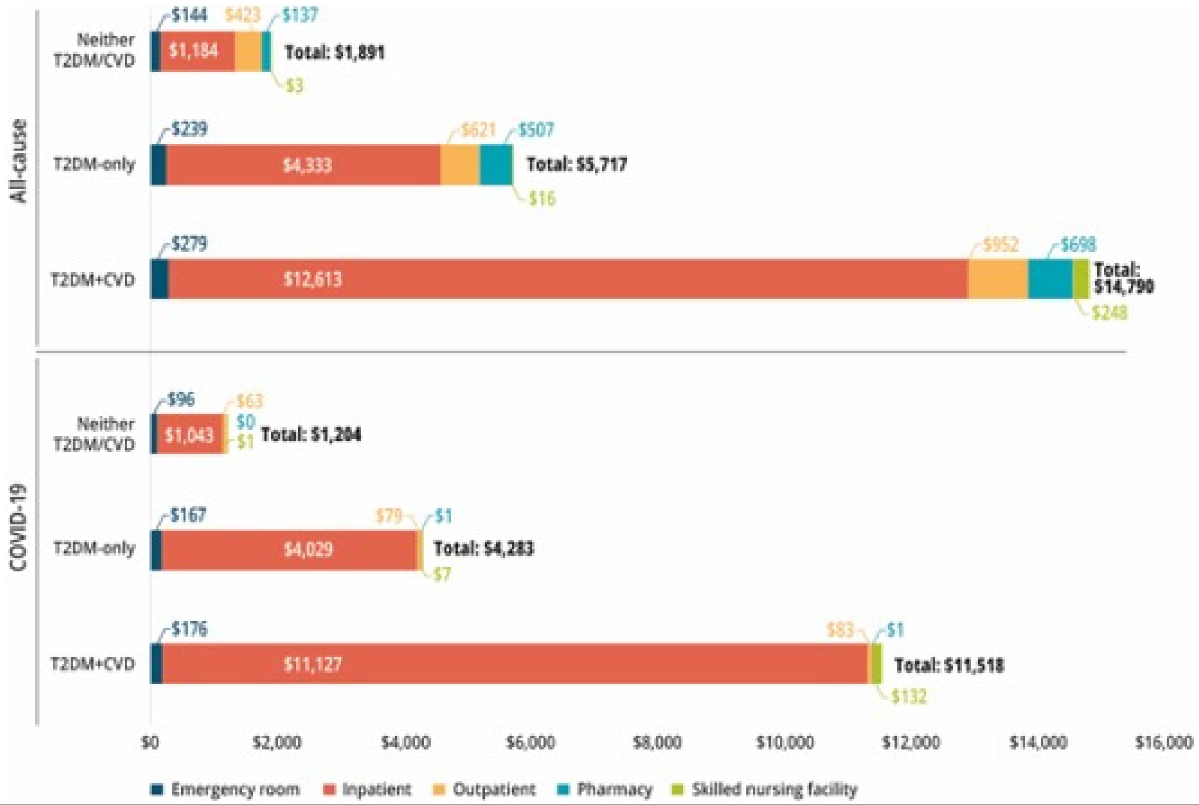

All *P* values <.001 for comparison of the 3 cohorts. Individual costs may not sum to total due to rounding.
Abbreviations: CVD, cardiovascular disease; PPPM, per patient per month; T2DM, type 2 diabetes mellitus.

Figure S3. Mean Monthly All-Cause Costs After COVID-19 Infection Before Propensity Score Matching

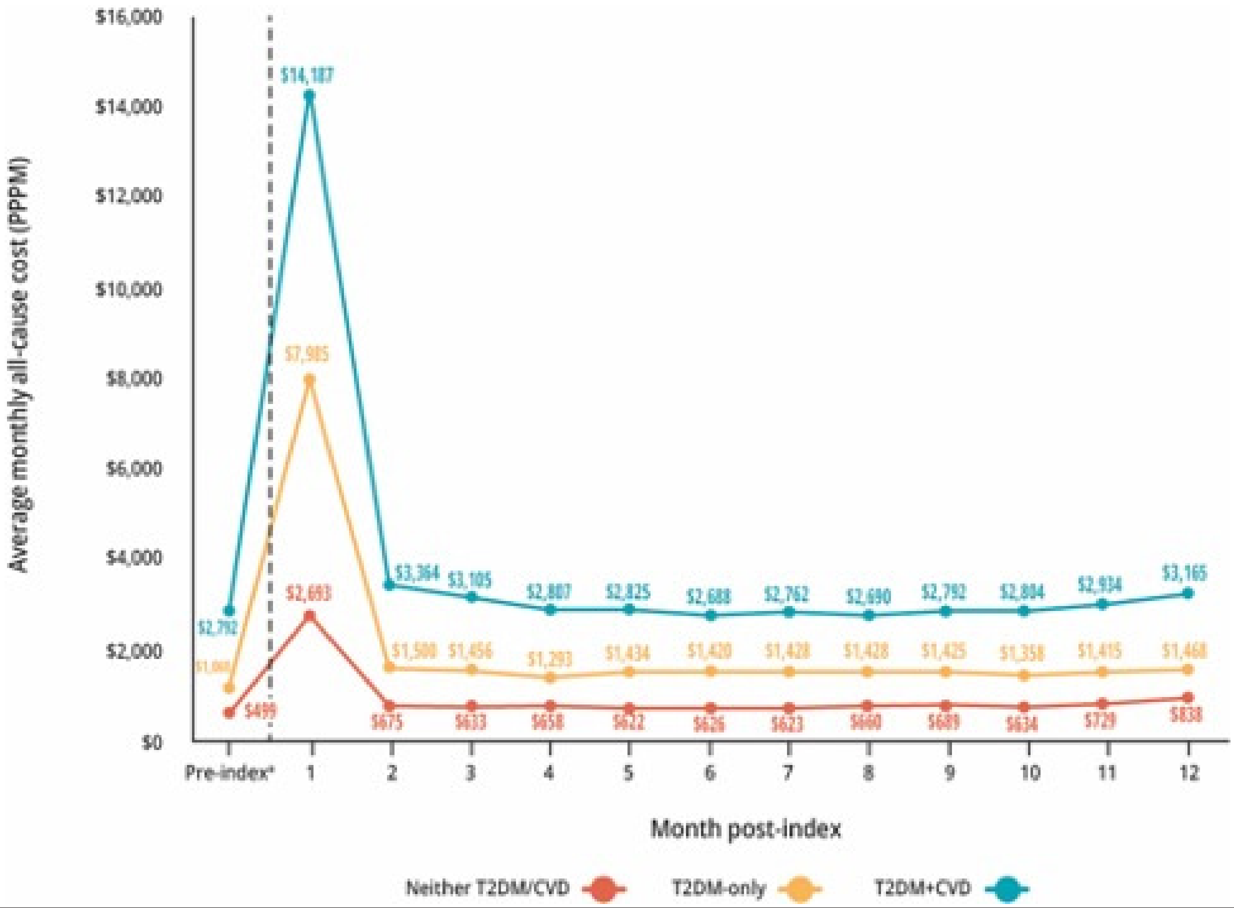

[a]Pre-index costs were calculated as the mean costs during the 12-month period before the index date; all *P* values <.05 for comparison of the 3 cohorts. *Vertical dotted line* represents the index date.

Abbreviations: CVD, cardiovascular disease; PPPM, per patient per month; T2DM, type 2 diabetes mellitus.
